# The effect of exercise on the cognitive and physical function of patients with dementia

**DOI:** 10.1192/j.eurpsy.2021.1034

**Published:** 2021-08-13

**Authors:** V. Papatsimpas, S. Vrouva, D. Bakalidou

**Affiliations:** 1 Physiotherapy Department, General Hospital Athens “G. Gennimatas”, Athens, Greece; 2 Physiotherapy Department, 401 General Military Hospital of Athens, Athens, Greece; 3 Department Physiotherapy, University of West Attica, Egaleo, Greece

**Keywords:** dementia, Exercise, cognitive function, physical function

## Abstract

**Introduction:**

Dementia is characterized by a decrease in mental functions, while disorders of balance, coordination of movements and gait are gradually added. In recent years there has been a growing interest in the role of exercise as a therapeutic strategy for people with dementia.

**Objectives:**

The aim of this study was to investigate the effect of different types of exercise and its parameters on cognitive and physical function in patients with dementia after reviewing the relevant literature.

**Methods:**

Review of the literature based on the research of original scientific articles published in the electronic databases PubMed / Medline and Google scholar using as keywords the terms dementia, cognitive function, physical function, functionality, aerobic exercise, resistance exercise.

**Results:**

A review in the literature highlights the beneficial effect of exercise on patients with dementia. Aerobic exercise and mixed interventions have been studied more, while resistance interventions have been less studied. All three types of exercise have shown positive effects. The methodology differences of the studies make it difficult to draw definitive conclusions about the optimal intervention in the cognitive and physical function for the optimal result, the type of exercise, the duration, the frequency and the intensity.

**Conclusions:**

Exercise (physical) may help maintain or improve cognitive function and functionality in patients with dementia but additional study is needed to clarify optimal intervention and establish guidelines.

